# A dementia care training using mobile e-learning with mentoring support for home care workers: a controlled study

**DOI:** 10.1186/s12877-021-02075-3

**Published:** 2021-02-16

**Authors:** Hsin-Feng Su, Malcolm Koo, Wen-Li Lee, Huei-Chuan Sung, Ru-Ping Lee, Wen-I Liu

**Affiliations:** 1 Department of Nursing, Tzu Chi University of Science and Technology, No. 880, Sec. 2, Chien-Kuo Road, Hualien City, 970046 Taiwan; 2 Institute of Medical Sciences, Tzu Chi University, 701 Zhongyang Road, Sec. 3, Hualien City, 97004 Taiwan; 3 Graduate Institute of Long-term Care, Tzu Chi University of Science and Technology, No. 880, Sec. 2, Chien-Kuo Road, Hualien City, 970046 Taiwan; 4 Department of Medical Image & Radiological Science, Tzu Chi University of Science and Technology, No. 880, Sec. 2, Chien-Kuo Road, Hualien City, 970046 Taiwan; 5 School of Nursing, National Taipei University of Nursing and Health Sciences, 365 Ming-te Road, Peitou District, Taipei City, 112 Taiwan

**Keywords:** Dementia, Online, Mobile learning, Home care, Peer support, Knowledge, Competence

## Abstract

**Background:**

Caring of older adults with dementia at home can be challenging for home care workers. There is a need to develop suitable training for home care workers to improve the quality of dementia care. We evaluated a 12-week dementia care training including mobile e-learning, social networking, and mentoring support group meetings on the dementia care knowledge, attitude, and competence of home care workers.

**Methods:**

This controlled study involved 140 home care workers from two home care agencies, which were selected from 12 home care agencies in eastern Taiwan. The two home care agencies were randomly allocated either the intervention group or the control group. The intervention group received mobile e-learning, mentor-led online social support networking, and monthly face-to-face mentoring support group meetings. Participants in the control group received 8-h conventional lectures. The primary outcomes were knowledge, attitude, and competence in dementia care. Questionnaires consisting of the Dementia Knowledge Assessment Scale, Approaches to Dementia Questionnaire, and Sense of Competence in Dementia Care Staff scale were administered to the participants at three time points (baseline, end of the 12-week intervention, and 12 weeks after the end of the intervention).

**Results:**

Generalized estimating equation analyses showed that the intervention significantly improved the knowledge, attitude, and competence of home care workers on dementia care. The effects remained significant even 12 weeks after the end of the intervention.

**Conclusions:**

A 12-week dementia care training program consisting of mobile e-learning, social networking, and face-to-face mentoring support group meetings were found to a feasible approach in improving the knowledge, attitude, and competence of home care workers. Mobile e-learning and online environment provides a platform that is self-directed, flexible, accessible, and cost-effective for training home care workers. The findings provide a call to action for nurse educators and policy makers to re-design existing dementia care training for home care workers to meet the critical home care needs of a growing dementia population.

**Trial registration:**

ClinicalTrials.gov. NCT03822286. Registration date: 27/01/2019. Posted date: 31/01/2019.

## Background

The rapid aging of the population is a challenge faced by many countries, and Taiwan is no exception. As the population ages, the number of people affected by dementia will increase accordingly [1]. Over 9.9 million new cases of dementia were anticipated each year worldwide, implying one new case every 3.2 s [2]. The number of people living with dementia worldwide is currently estimated at 50 million and will almost triple by 2050 [3]. There are currently 0.29 million people living with dementia in Taiwan. The numbers are projected to reach 0.67 million by 2030 and to exceed 0.88 million by 2050 [4]. Dementia is a costly illness that puts a heavy strain on health care infrastructure, including tremendous demands on formal and informal caregivers [5, 6].

Dementia is a syndrome of cognitive decline, including deterioration in memory, thinking, behavior, and the ability to perform everyday activities. Patients with dementia are often accompanied by multiple chronic illnesses that require complex nursing care needs [7]. Dementia can negatively impact the physical, psychological, social wellbeing, and economy, not only on people with dementia, but also on their caregivers, families, and society [3]. Providing care to a family member with dementia can severely affect the physical, emotional, and social health of family caregivers [8]. With the launch of the long-term care plan version 2.0 in November 2016 in Taiwan, there is improved support for home care services and dementia patients [9]. The rapidly growing population of older adults with the majority of those with dementia residing at homes will drive demand for home care workers even higher. Recruiting adequate numbers of home care workers to fill these jobs has becoming increasingly difficult, as evidenced by continual reports of workforce shortages. One reason for the shortages is the poor quality of home care work conditions. Home care workers are undervalued and underpaid. The majority of them receives inadequate training and often has to deal with erratic work schedule due to varied client care needs. A study found that home care workers recognized the needs to work together with other health care professionals as a team, and adequate training is needed to improve care outcomes of those with dementia, such as nutritional care [10].

The home care workforce in Taiwan primarily comprised of middle-aged women with the majority have completed no formal education beyond high school and received insufficient training on dementia care. A survey study on 407 home care workers and 30 supervisors in Taiwan revealed that home care staff received a mean 37.5 h of training a year. The type of training is mainly in-house educational training without specific programs on dementia care [11]. In addition, due to heavy work schedule and time constraints, in-person training programs held during non-working hours are difficult for home care workers to attend [12]. Therefore, other approaches for training with consideration of the typical characteristics of home care workers in Taiwan are needed.

A recent approach to provide staff development with a flexible schedule is mobile e-learning [13]. Training materials can be made available online as an e-book, and home care staff can access them using a mobile phone or tablet at their convenience [14]. In addition to e-book, the use of social networking platforms can be combined with mobile e-learning. These online platforms can provide learners with a virtual space that encourages active learning through both synchronous and asynchronous peer discussions. Users can also benefit from cooperative learning, which has been linked to increased levels of student satisfaction [15] and perceived social support [16]. A systematic review of Internet-based supportive interventions for caregivers of patients with dementia revealed that these interventions could improve various aspects of caregiver well-being, such as confidence, depression, and self-efficacy, when they were comprised of multiple components and were tailored to the individual [17]. In addition, a study on 40 certified nurse assistants found that an Internet-based training module on dementia care was a suitable method to deliver dementia care training, and was able to improve the dementia care knowledge of nursing home nurse assistants [18].

However, to the best of our knowledge, no studies have yet focused on the use of mobile e-learning combining with a social networking mentoring support for dementia care training on home care workers. Therefore, the aim of the present study was to evaluate a 12-week mobile e-learning dementia care training with mentoring support using a social networking platform and monthly face-to-face support group meetings in improving the knowledge, attitude, and competence of home care workers in Taiwan.

## Methods

### Study design and participants

This pre/post/follow-up controlled study employed repeated measures, obtaining data at baseline and at 12- and 24-week post intervention. The study was conducted between January 2019 and October 2019 in eastern Taiwan. It was registered in the ClinicalTrials.gov with the identifier (NCT03822286). The Consolidated Standards of Reporting Trials (CONSORT) statement for the study design and reporting was adopted [19]. Using G*Power with an effect size of 0.25, two-tailed alpha of 0.05, and a power of 95%, the total sample size was estimated to be 120. Considering a 15% attrition rate, at least 70 participants in each group would be required in each group, with a total sample size of 140.

At the time of participant recruitment, there were a total of 12 home care agencies in eastern Taiwan. To meet our sample size requirement, the inclusion criterion was set to include only agencies with at least 70 home care workers. Of the 12 home care agencies, only two met the inclusion criteria. They were selected and randomized assigned to either the intervention group or the control group by an independent research assistant who was not involved with recruitment. Allocation assignment was concealed from the enrolling researcher.

The inclusion criteria for the participants included: (1) age 20 to 65 years, (2) Taiwanese nationality, (3) had worked as a home care worker for at least 3 months, (4) had experience in providing home care for patients with cognitive impairment or dementia, (5) had a mobile phone or tablet and regular access to the Internet.

### Intervention group

Participants allocated to the intervention group received a 12-week dementia care training, consisting of mobile e-learning material with mentor-led support group networking and three monthly face-to-face support group meetings. First, a mobile e-learning course, which was designed to be accessed on mobile phones or tablets, contained videos for dementia care on eight main modules. The topics of the modules were as follows: (1) psycho-behavioral symptom management skills, (2) communication skills, (3) cognitive and emotional assessment, (4) common dementia care problems and care skills, (5) needs assessment and health educational skills for families with dementia, (6) overview of therapeutic activities, (7) care skills for various stages of dementia, and (8) oral care for older adults with dementia. Each module was designed to be completed in 15 to 20 min. The e-learning course material could be accessed through an e-book app, and the material could be read online or offline, as preferred. Moreover, the course content was designed with a consideration of the educational level of typical home care workers in Taiwan. Colloquial style of writing, lively animations, dominance of images over text, and contextual videos were used to enhance the motivation of learning. The interface was simple and any part of the course content could be repeated as needed. Furthermore, online quizzes were available for learners to gauge their understanding of the content. Weekly reminders and a module completion checklist were used by mentors to ensure that all participants had completed each of the eight modules of the e-learning course.

The second component of the dementia care training was a mentor-led online support networking. One home care service supervisor would act as a mentor to lead 8 to 10 home care workers. The mentor would provide support over the phone or through the group chat function of the free social networking app LINE (https://linecorp.com/en/) on a daily basis. Home care workers could also discuss care problems, and receive prompt advice from senior mentors through LINE. New dementia care information and learning videos were uploaded frequently to the social networking platform to keep home care workers up-to-date with dementia care knowledge.

The third component of the dementia care training was monthly face-to-face support group meetings. Mentors and the home care workers met in person in the agency’s meeting room once a month for an hour to discuss issues and to share their experience in caring skills. Mentors would provide suggestions for strategies, encouragement and psychological support according to the problems and difficulties encountered by the home care workers in order to strengthen their dementia care skills and competence.

### The control group

Participants in the control group only received an 8-h of usual conventional instructor-led lectures on dementia care. They did not receive mobile learning, online support networking, or support group meetings.

### Outcome measures

Participants were asked to complete a paper-based questionnaire consisting of basic demographic characteristic and three measurement scales at three time points: (1) baseline [T_0_], (2) end of the 12-week intervention [T_1_], and (3) 12 weeks after the end of the intervention [T_2_]. The three scales were Dementia Knowledge Assessment Scale (DKAS), Approaches to Dementia Questionnaire (ADQ), and Sense of Competence in Dementia Care Staff scale (SCIDS) for assessing the participants’ knowledge, attitudes, and competence, respectively.

The DKAS contains 25 items with a maximum score of 50. A higher score represents better knowledge in dementia care. The original scale in English showed an overall Cronbach’s α of 0.85 when applied to a sample of 3649 people consisting of health professionals, students, family members of people with dementia, and the public. Confirmatory factor analysis showed good model fit with four clearly discernible factors [20]. The Cronbach’s α of the traditional Chinese version was 0.74 [21]. The Cronbach’s α of the DKAS was 0.73 in the present study.

The ADQ contains 19 items based on a 5-point Likert response that aim to capture the attitudes towards people with dementia. Possible scores for the ADQ range from 19 to 95, with higher scores indicative of more positive attitudes towards people with dementia [22]. The Chinese version was developed by Leung et al. [23]. The Cronbach’s α was found to be 0.79 in a study on nurse aides [24]. The Cronbach’s α of ADQ was 0.72 in the present study.

The SCIDS consists of 17 items for measuring the sense of competence in dementia care staff. Possible scores for the SCIDS range from 17 to 68, with higher scores indicative of better perceived abilities and skills in dementia care. When applied to a sample of 211 staff in dementia care in the United Kingdom, a good internal consistency (Cronbach’s α = 0.91) was obtained. The test-retest reliability measured by intra-class correlation was 0.74 [25]. The Cronbach’s α was 0.91 in the present study.

### Data collection

This study was conducted in accordance with the Helsinki Declaration [26] and the research protocol was approved by the institutional review board of the Ministry of Health and Welfare, Yuli Hospital, Taiwan (approval number: YLH-IRB-10721). Potential participants were invited with detailed instruction of study aims and procedures. All participants in this study signed written informed consent prior to enrollment. The participants were informed that they were free to withdraw during the study at any time, for any reason, without any need for explanation, and that the confidentiality of the data sets would be maintained. After written consent was obtained, participants’ baseline data were collected. The baseline and follow-up data at 12-week and 24-week were assessed by a trained research assistant.

### Statistical analysis

All statistical analyses were performed with IBM SPSS Statistics for Windows, Version 25.0.0.1 (Armonk, New York, USA). The characteristics of the study participants between groups at baseline were compared with t-test for continuous data and Chi-square test or Fisher’s exact test, as appropriate, for nominal data. We also calculated effect sizes with Cohen’s *d* by dividing the mean difference between the two groups by the pooled standard deviation at T_1_ and T_2_. According to Cohen, a *d* = 0.2, 0.5, and 0.8 could be considered as a small, medium, and large effect size, respectively [27]. In addition, general estimating equation (GEE) was used to assess the group × time interaction effect, that is, to compare the changes in the outcome variables between the two study groups over the three time points. An unstructured working correlation matrix was used to model the within-subject error correlation structure. Baseline variables with a *p* value of < 0.20 were included in the GEE analyses to evaluate their potential confounding effects. The criterion for significance was set at *p* < 0.05.

## Results

**Fig. 1:**
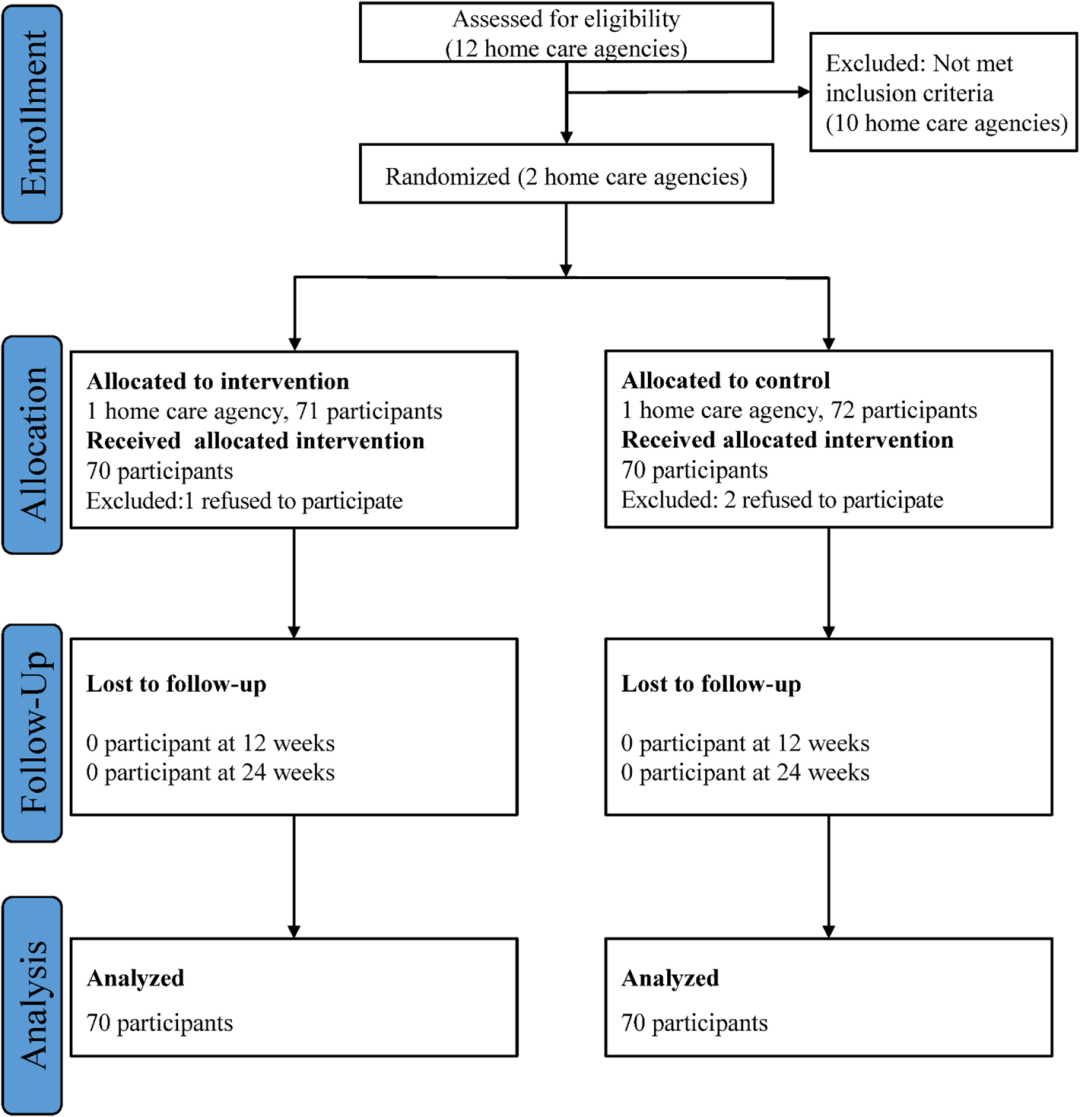
CONSORT flow diagram

There were a total of 143 home care workers eligible for the study with 71 in the intervention group and 72 in the control group. One person refused to participate in the intervention group and two people refused in the control group. Therefore, 70 home care workers in each group completed the study. The study flow diagram is presented in Fig. 1. The characteristics of the study participants at baseline are shown in Table 1. The overall mean age was 47.8 years and most participants (98.6%) were female. Educational level and on-the-job training in dementia care in the previous year were significantly different between the two groups.

**Table 1:** Demographic characteristics of study participants (*N* = 140)

Variable	All *(N* = 140)	Intervention group (*n* = 70)	Control group (*n* = 70)	*p*
Age, year, mean (SD)	47.8 (7.9)	47.3 (7.1)	48.4 (8.7)	0.396
Sex				> 0.999
female	138 (98.6)	69 (98.6)	69 (98.6)	
male	2 (1.4)	1 (1.4)	1 (1.4)	
Marital status				0.624
being married	92 (65.7)	43 (61.4)	49 (70.0)	
divorced	22 (15.7)	13 (18.6)	9 (12.9)	
widowed	20 (14.3)	10 (14.3)	10 (14.3)	
unmarried	6 (4.3)	4 (5.7)	2 (2.9)	
Educational level				0.001
elementary school	15 (10.7)	8 (11.4)	7 (10.0)	
junior high school	38 (27.1)	9 (12.9)	29 (41.4)	
senior high school and vocational school	72 (51.4)	45 (64.3)	27 (38.6)	
university	15 (10.7)	8 (11.4)	7 (10.0)	
Job type				0.562
full-time	127 (90.7)	65 (92.9)	62 (88.6)	
part-time	13 (9.3)	5 (7.1)	8 (11.4)	
Years worked as a home care worker, mean (SD)	8.8 (5.3)	8.8 (5.1)	8.7 (5.5)	0.868
On-the-job educational training in previous year, hours				0.098
None	1 (0.7)	0 (0)	1 (1.4)	
< 10	5 (3.6)	5 (7.1)	0 (0)	
10–20	25 (17.9)	12 (17.1)	13 (18.6)	
> 20	109 (77.9)	53 (75.7)	56 (80.0)	
On-the-job educational training on dementia in previous year, hours				0.002
None	15 (10.7)	5 (7.1)	10 (14.3)	
< 2	21 (15.0)	13 (18.6)	8 (11.4)	
3–6	25 (17.9)	12 (17.1)	13 (18.6)	
7–12	30 (21.4)	23 (32.9)	7 (10.0)	
> 12	49 (35.0)	17 (24.3)	32 (45.7)	

Data are n (%), unless otherwise stated. SD: standard deviation

**Fig. 2:**
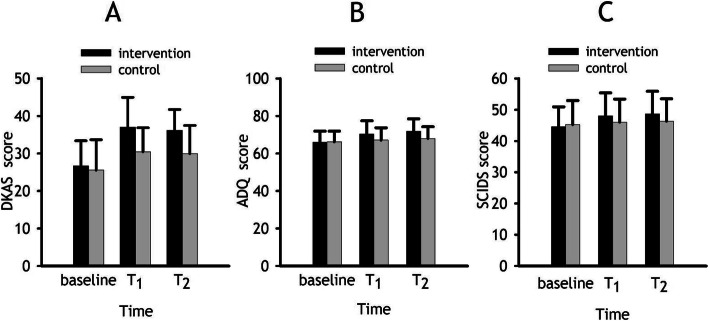
Bar graphs showing mean scores of the three outcome variables (**a**. DKAS: Dementia Knowledge Assessment Scale; **b**, ADQ: Approach to Dementia Questionnaire; **c**. SCIDS: Sense of Competence in Dementia Care Staff scale) for the intervention group and the control group at baseline, week 12 (T_1_), and week 24 (T_2_). Error bars represent standard deviation

In addition, three outcome variables (DKAS, ADQ, and SCIDS) were not significantly different between the two groups at the baseline. The descriptive statistics of the three outcome variables between the two groups at baseline (T_0_), week 12 (T_1_), and week 24 (T_2_) are shown in Table 2 and Fig. 2. At baseline, there were no statistically significant differences in the scores of all three scales between the two groups. At week 12 (T_1_) and week 24 (T_2_), the DKAS and ADQ scores were significantly higher in the intervention group compared with the control group.

**Table 2:** Descriptive statistics of the scores of DKAS, ADQ, and SCIDS between the two groups at baseline, the end of the intervention and 12 weeks post-intervention

Variable	Time	Group, mean (SD)		*p*
		Intervention (*n =* 70)	Control (*n* = 70)	
DKAS score	baseline	26.69 (6.72)	25.54 (8.14)	0.367
	T_1_	37.04 (7.93)	30.43 (6.44)	< 0.001
	T_2_	36.20 (5.55)	29.94 (7.54)	< 0.001
ADQ score	baseline	66.00 (5.94)	66.23 (5.70)	0.817
	T_1_	70.34 (7.15)	67.14 (6.59)	0.007
	T_2_	71.87 (6.61)	68.04 (6.22)	0.001
SCIDS score	baseline	44.61 (6.33)	45.23 (7.73)	0.608
	T_1_	48.00 (7.40)	46.01 (7.41)	0.115
	T_2_	48.69 (7.22)	46.36 (7.16)	0.057

*DKAS* Dementia Knowledge Assessment Scale, *ADQ* Approach to Dementia Questionnaire, *SCIDS* Sense of Competence in Dementia Care Staff scale, *SD* Standard deviation, *T*_*1*_ end of the intervention; T_2_ = 12 weeks post-intervention

Results from the GEE analysis indicated that the group by time interaction were significant in all three outcome variables at both T_1_ (DKAS, *p* < 0.001; ADQ, *p* = 0.003; and SCIDS, *p* = 0.043) and T_2_ (DKAS, *p* < 0.001; ADQ, *p* = 0.001; and SCIDS, *p* = 0.020) (Table 3). The parameter estimates were all positive at both time points indicated that DKAS scores, ADQ scores, and SCIDS scores were significantly higher in the intervention group compared with the control group.

**Table 3:** General estimating equation (GEE) analysis of the scores on Dementia Knowledge Assessment Scale (DKAS), Approach to Dementia Questionnaire (ADQ), and Sense of Competence in Dementia Care Staff scale (SCIDS) for the intervention and control group

Variable	Dementia Knowledge Assessment Scale (DKAS)		Approach to Dementia Questionnaire (ADQ)		Sense of Competence in Dementia Care Staff scale (SCIDS)	
	β (95% CI)	*p*	β (95% CI)	*p*	β (95% CI)	*p*
Group (intervention)^a^	0.36 (−2.33–3.04)	0.796	−0.28 (−2.60–2.04)	0.813	−1.29 (−3.58–1.01)	0.272
Time^b^						
T_1_	4.89 (3.03–6.74)	< 0.001	0.91 (−0.52–2.35)	0.213	0.79 (−0.95–2.52)	0.375
T_2_	4.40 (2.58–6.22)	< 0.001	1.81 (0.30–3.32)	0.019	3.09 (− 0.45–2.71)	0.162
Group × time^c^						
Intervention × T_1_	5.47 (2.92–8.02)	< 0.001	3.43 (1.18–5.68)	0.003	2.60 (0.08–5.12)	0.043
Intervention × T_2_	5.11 (2.70–7.53)	< 0.001	4.06 (1.62–6.50)	0.001	2.94 (0.47–5.42)	0.020

*β* parameter estimate, *CI* Confidence interval, *T*_*1*_ end of the intervention, *T*_*2*_ 12 weeks post-intervention

^a^Reference group = control group

^b^Reference group = baseline (T_0_)

^c^Reference group = intervention × T_0_

Three baseline variables, including educational levels, on-the-job educational training in previous year, and on-the-job educational training on dementia in previous year, were adjusted in the general estimating equation analysis

The effect size for DKAS, ADQ, and SCIDS at T_1_ and T_2_ are shown in Table 4. The effect size for DKAS was large at both T_1_ (0.91) and T_2_ (0.94). The effect size for ADQ was medium at both T_1_ (0.46) and T_2_ (0.60). However, for SCIDS, the effect size was only small to medium at both T_1_ (0.27) and T_2_ (0.32).

**Table 4:** Effect size of the scores on Dementia Knowledge Assessment Scale (DKAS), Approach to Dementia Questionnaire (ADQ), and Sense of Competence in Dementia Care Staff scale (SCIDS) at the end of the intervention (T_1_) and 12 weeks post-intervention (T_2_)

Variable	Cohen’s *d* (95% confidence interval)	
	T_1_	T_2_
DKAS	0.91 (0.57–1.26)	0.94 (0.59–1.29)
ADQ	0.46 (0.13–0.80)	0.60 (0.26–0.93)
SCIDS	0.27 (−0.07–0.60)	0.32 (−0.01–0.66)

*T*_*1*_ End of the intervention, *T*_*2*_ 12 weeks post-intervention

## Discussion

The study showed that a 12-week dementia care training consisting of mobile e-learning, mentor-led online support networking, and face-to-face mentoring support group meetings could significantly improve dementia care knowledge, attitude, and competence of home care workers at the end of the intervention, and the effects remained at 24 weeks post-intervention. A comparison of effect sizes across the three outcomes showed that the intervention was most effective in improving dementia care knowledge, followed by attitude. Improvement in perceived competence, while statistically significant, was modest in comparison. A possible reason may be that it took a longer time for home care workers to feel confident in their caring skills because of the complex home care environment and a variety of care challenges of community-dwelling persons living with dementia. A few home care workers reported that they lacked experience in handling emergent issues at the person’s homes, which made them feel frustrated and non-confident.

To the best of our knowledge, this study was the first to demonstrate a beneficial effect of dementia care training combining mobile e-learning with mentoring support on dementia care knowledge, attitude, and competence in home care workers caring for community-dwelling patients with dementia. In addition, our results were consistent with other studies of Internet-based training on dementia care. A study comprising 105 Taiwanese nurses found that a 16-week internet-based communication education program was able to significantly improve nurses’ communication knowledge, frequency in assessing dementia patients’ communication capacity, and communication performance [28]. Another study with nursing home nurse assistants found that an Internet-based training was a time- and cost-efficient strategy to deliver dementia care training, and able to improve dementia care knowledge of nursing home care staff [18]. However, none of these studies used mentoring support in addition to Internet-based training, and none focused on home care workers.

“Aging in place” is described as a person remains living in the place of their choice in the community with some level of independence and autonomy [29], is an important initiative of long-term care policy in many parts of the world, including Taiwan. Having people remains in their homes as long as possible can avoid the costly option of institutional care and allow them to live with dignity in their own communities. To achieve this objective, not only an increasing number of home care staff is necessary, the amount and type of in-service training to meet the long-term care needs of individuals with various illnesses, such as behavioral and psychological symptoms of patients with dementia, will also be in demand [30]. It was suggested that the training which focused on the management of behavioral and psychological symptoms could have greatest positive impact on staff sense of competence [31]. Staff training has been shown to be associated with improved patient outcomes [32], and it could also exert beneficial effects on the health of home care workers themselves. A study of 29 home care assistants in Spain reported that a program with 13 weekly training sessions was able to reduce their dysfunctional thoughts about care, which could be acted as mediators in caregiving stress [33]. Furthermore, a study on 25 hospital social workers found that professional skill-development training was able to alleviate burnout [34]. This may have important implications for job satisfaction and staff retention. Despite these potential advantages of staff training, finding time to attend training courses is one of the common barriers for home care workers with heavy workload and tight schedule.

The present study showed that mobile e-learning with mentor-led online support networking might be able to address this administrative barrier. Home care workers could access the dementia care e-learning materials on their mobile phones or tablets at their convenience. Internet access via mobile phones is inexpensive and widely available in Taiwan. Home care workers could also discuss care problems, and receive prompt advice and support from senior mentors through the LINE social networking platform. The home care workers therefore did not feel alone while taking care of dementia patients at home settings. New dementia care information and learning videos were frequently uploaded to the social networking platform to keep workers up-to-date with dementia care knowledge.

More than half of our study participants had completed no formal education beyond senior high school. The design of e-learning materials with a consideration of the educational level of typical home care workers in Taiwan was important. Colloquial style of writing, simple interface, lively animations, dominance of images over text, and contextual videos could enhance the learning motivation of the home care workers. At the completion of the third group meeting, many participants commented that the mobile e-learning materials were easy to understand, and attracted their attention to read and learn compared to that of traditional didactic courses. Therefore, the knowledge, attitude, and competence of home care workers were significantly improved with this approach compared with traditional face-to-face training courses. However, a few older home care workers mentioned that it took them some time to successfully access the e-learning materials due to unfamiliarity in installing an app on their mobile phone. Moreover, a few participants experienced lagging and temporary interruptions of Internet connection while downloading e-learning materials because of unstable Internet connection when they were in rural mountain areas.

Monthly face-to-face support group meetings could provide a channel for senior mentors and home care workers to exchange dementia care experiences and to come up with better care strategies through brainstorming and discussion sessions. In addition, regular support group meetings also provide opportunities for workers to support each other emotionally and to form better team bonding and cohesiveness. There were no unintended or adverse effects occurred in this study.

Regarding the participation rates at the three measurement time points, only five participants (7.1%) not attend all three support group meetings due to conflicting work schedule or personal sickness. All participants were able to attend at least two group meetings. Several reasons could explain the good participation rates. First, we sent advanced reminders using LINE before each meeting. Second, small gifts and snacks were provided at each meeting. Third, and probably the most important incentive is that participants would receive continuing education credits for attending the meetings.

There are a number of limitations we wish to highlight that could inform future research. First, the study population was restricted to home care workers in two home care service agencies in eastern Taiwan which limits the generalisability of the study findings. For example, there were some differences between two agencies, such as management hierarchical structures and one of them was located in an urban area whereas the other was located in a rural area. Nevertheless, the two home care agencies had many similar characteristics. They were both private organizations that provide home care and home nursing services. The work hours, work schedules, and wages of the home care workers are similarly governed by labor regulations. In addition, we adjusted for the potential confounding effects of educational level and previous on-the-job educational training of the participants by including these variables in the GEE analyses. Second, the actual time spent accessing the mobile e-learning course for each participant and the time spent using the mentor-led online support networking were not available and therefore, we were unable to assess the relative contribution for these two components on the observed effect. Third, the individual contributions from e-learning, social networking among peers, and mentor support could not be evaluated. Fourth, it is not possible to blind a participant to his or her group allocation, and the outcomes were based on subjective self-reported measurements, which might be influenced by the Hawthorne effect. Fifth, the researcher acted as the facilitator for the face-to-face mentoring support group meetings and therefore, might have inadvertently affected the quality of the intervention delivery. Finally, we did not conduct any formal assessment on the opinion of the participants regarding the content of the course materials. Nevertheless, as mentioned above, many participants mentioned that the mobile e-learning materials were easy to understand, and were able to attract their attention to read and learn.

## Conclusion

With increasing needs for home care services in Taiwan, the demand for home care workers is expected to rise dramatically in coming years. It becomes increasingly more important to provide appropriate benefits, better working conditions, and adequate training for home care workers to improve their quality of care services and job retention. Our study results revealed that a 12-week dementia care training consisting of mobile e-learning, mentor-led online support networking, and monthly face-to-face mentoring support group meetings is an innovative and promising training approach to improve dementia care knowledge, attitude, and competence of home care workers. Mobile e-learning and online environment provides a platform that is self-directed, individualized, interactive, flexible, accessible, and cost-effective for home care workers. The findings provide a call to action for nurse educators and home care agencies to coordinate with policy development efforts in re-designing existing dementia care training for home care workers so as to meet the critical home care needs of a growing dementia population.

## Data Availability

The datasets used and/or analyzed during the current study are available from the corresponding author on reasonable request.

## Abbreviations

ADQ: Approaches to Dementia Questionnaire
CI: Confidence interval
DKAS: Dementia Knowledge Assessment Scale
GEE: General estimating equation
SCIDS: Sense of Competence in Dementia Care Staff scale
SD: Standard deviation
